# RURALITY MATTERS: UNDERSTANDING THE CRITICAL AND NUANCED ROLE OF PLACE-BASED FACTORS IN RURAL AGING

**DOI:** 10.1093/geroni/igae098.1818

**Published:** 2024-12-31

**Authors:** Jessica Cassidy, Steven Cohen, Leif Jensen

**Affiliations:** Chair; Co-Chair; Discussant

## Abstract

Although only 15% of all Americans live in rural communities, 22% of U.S. older adults live in rural areas. Despite the prevalence of rural older adults, there are significant gaps in the gerontological literature regarding this vulnerable sub-population and furthermore, the multidimensional factors that characterize rurality and influence their healthy aging. To highlight the impact of place-based factors on the breadth of needs and outcomes found in rural aging populations, this symposium will host five research projects spanning the social and health sciences. The first study applies multi-level models to estimate the spatial distribution of aging and disability service organizations by sociodemographic and sociocultural characteristics in U.S. rural counties. The second uses multiple methods to contrast challenges facing older adults aging in place in rural areas with assets and opportunities for supporting healthy aging in rural older adults. The third examines rural-urban differences in health outcomes and the lived experiences of informal caregivers to suggest culturally appropriate intervention strategies, tailored to the needs of rural caregivers. The fourth analysis compares differences in rural health disparities among diverse aging populations living in rural Wyoming and Mississippi. The fifth investigates benefits of examining claims versus survey data when comparing rural and urban areas to better understand access to healthcare services. Our discussant will lead conversation to emphasize the important nuances of healthy aging in rural communities and to increase awareness on research and policy strategies that may support the needs of older adults and caregivers living in rural communities.

